# Supplementary material for: GermVarX: A Robust Workflow for Joint Germline Variant Exploration in whole-exome sequencing cohorts
Source: PLoS One. 2026 Apr 2;21(4):e0345561. doi: 10.1371/journal.pone.0345561 (PMC13046259; doi:10.1371/journal.pone.0345561)
Supplement: S1 File — (PDF) [file pone.0345561.s001.pdf]

Mar 10, 2026 Version 1

GermVarX: An Automated Workflow for Joint Germline Variant Exploration in Whole-Exome Sequencing Cohorts V.1

DOI
https://dx.doi.org/10.17504/protocols.io.3byl48kr8vo5/v1

Nguyen Thi Phuong Thao[1], Nguyen Duc Dung[1], Mai Van Thuy[2], Nguyen Khoi Dung[3], Nguyen Dang Tung[4], Truong Thi Minh Ngoc[1], Ha Hong Hanh[5], Tran Thi Ha Trang[6]

[1]Institute of Information Technology, Vietnam Academy of Science and Technology, Hanoi, Vietnam;
[2]Hanoi University of Public Health, Hanoi, Vietnam; [3]Electric Power University, Hanoi, Vietnam;
[4]Post and Telecommunications Institute of Technology, Hanoi, Vietnam;
[5]Institute of Biology, Vietnam Academy of Science and Technology, Hanoi, Vietnam;
[6]VinUni Bigdata Research Institute, VinUniversity, Hanoi, Vietnam

Nguyen Thi Phuong Thao: Corresponding author;

Nguyen Thi Phuong Thao

Create & collaborate more with a free account

Edit and publish protocols, collaborate in communities, share insights through comments, and track progress with run records.

Create free account

OPEN ACCESS

DOI: https://dx.doi.org/10.17504/protocols.io.3byl48kr8vo5/v1

Protocol Citation: Nguyen Thi Phuong Thao, Nguyen Duc Dung, Mai Van Thuy, Nguyen Khoi Dung, Nguyen Dang Tung, Truong Thi Minh Ngoc, Ha Hong Hanh, Tran Thi Ha Trang 2026. GermVarX: An Automated Workflow for Joint Germline Variant Exploration in Whole-Exome Sequencing Cohorts. **protocols.io**
https://dx.doi.org/10.17504/protocols.io.3byl48kr8vo5/v1 Version created by **Nguyen Thi Phuong Thao**

Manuscript citation:
PONE-D-25-50266

License: This is an open access protocol distributed under the terms of the **Creative Commons Attribution License**, which permits unrestricted use, distribution, and reproduction in any medium, provided the original author and source are credited

Protocol status: Working
We use this protocol and it's working

Created: January 20, 2026

Last Modified: March 10, 2026

Protocol Integer ID: 238948

Keywords: Bioinformatics Pipeline, Automated Workflow, Whole-Exome Sequencing, Germline Variants, Joint Variant Calling, Cohort Analysis, source workflow for joint germline variant discovery, automated workflow for joint germline variant exploration, exome sequencing cohorts germvarx, joint germline variant discovery, joint germline variant exploration, key feature of germvarx, germvarx, enabling simultaneous genotyping, simultaneous genotyping of multiple sample, gatk haplotypecaller, joint genotyping, variant effect predictor, implementation of joint variant calling, cohort, joint variant calling, task execution across diverse computing environment, downstream analysis, variant caller, wes cohort study, unified reporting, exploration in wes cohort study, automated workflow

Funders Acknowledgements:
Vietnam Ministry of Science and Technology
Grant ID: KC4.0-37/19-25
Vietnam Academy of Science and Technology
Grant ID: CSCL02.06/24-25

Abstract

GermVarX is an open-source workflow for joint germline variant discovery and exploration in WES cohort studies. A key feature of GermVarX is its implementation of joint variant calling, enabling simultaneous genotyping of multiple samples to produce a single, high-confidence multi-sample VCF, optimized for downstream analyses. Implemented in Nextflow DSL2 with Docker, it supports fully automated execution, a modular architecture, and parallelized task execution across diverse computing environments, including workstations, HPC clusters, and cloud platforms. The workflow integrates two state-of-the-art variant callers—GATK HaplotypeCaller and DeepVariant—with joint genotyping performed via GATK or GLnexus. To increase reliability, GermVarX supports consensus generation between callers, coupled with sample- and cohort-level quality control, functional annotation using the Variant Effect Predictor (VEP), and unified reporting through MultiQC. In addition, it provides PLINK-compatible outputs, facilitating seamless integration with statistical and association analyses.

Troubleshooting

Prepare the Computational Environment

1 GermVarX is distributed as a Nextflow pipeline with Docker container support.

To set up the environment:

1.1 **Install Docker**

Follow the installation instructions for your platform:

https://docs.docker.com/engine/install/.

1.2 **Install Nextflow**

GermVarX requires Nextflow (version ≥ 24).

Installation instructions:**https://www.nextflow.io/docs/latest/getstarted.html.**

Download the GermVarX Pipeline

2 Clone the source code from the official GitHub repository:

```
git clone https://github.com/thaontp711/GermVarX.git
cd GermVarX
```

Set Up Docker Images

3 Pull the required pre-built images and build the GermVarX custom image:

```
# PLINK 1.9
docker pull quay.io/biocontainers/plink:1.90b6.21--h516909a_0
```

```
# GATK 4.2.6.1
docker pull broadinstitute/gatk:4.2.6.1
```

```
# DeepVariant 1.6.1
docker pull google/deepvariant:1.6.1
```

```
# VEP 114.1
docker pull ensemblorg/ensembl-vep:release_114.1
```

```
# GLnexus 1.4.1
docker pull quay.io/biocontainers/glnexus:1.4.1--h17e8430_5
```

```
# GermVarX pipeline (custom image)
docker build -t germvarx-pipeline:0.1 ./docker/germvarx-pipeline
```

Prepare Testing Data and Resources

4 **Testing data**

Create a directory for the test data and download paired-end WES FASTQ files for two samples along with the corresponding target BED file:

```
mkdir -p testdata/fastq testdata/bed
cd testdata/fastq

# Sample 1: NA12891
wget https://storage.googleapis.com/brain-genomics-public/research/sequencing/fastq/novaseq/wes_agilent/50x/NA12891.novaseq.wes_agilent.50x.R1.fastq.gz
wget https://storage.googleapis.com/brain-genomics-public/research/sequencing/fastq/novaseq/wes_agilent/50x/NA12891.novaseq.wes_agilent.50x.R2.fastq.gz
# Sample 2: NA12892
wget https://storage.googleapis.com/brain-genomics-public/research/sequencing/fastq/novaseq/wes_agilent/50x/NA12892.novaseq.wes_agilent.50x.R1.fastq.gz
wget https://storage.googleapis.com/brain-genomics-public/research/sequencing/fastq/novaseq/wes_agilent/50x/NA12892.novaseq.wes_agilent.50x.R2.fastq.gz
cd ../bed
wget https://storage.googleapis.com/brain-genomics-public/research/sequencing/grch38/bed/agilent.targets.grch38.bed
```

5 **Reference resources**

Create a directory (e.g., named ReferenceDir) and download the required reference files as listed below. Some databases (e.g., dbSNP, dbNSFP, gnomAD, CADD) have multiple releases; you may choose an alternative version depending on your analysis preference and compatibility requirements. Be sure to download the corresponding index file as well.

- Reference genome (GRCh38): https://storage.googleapis.com/gcp-public-data--broad-references/hg38/v0/Homo_sapiens_assembly38.fasta
- dbSNP 138: https://storage.googleapis.com/genomics-public-data/resources/broad/hg38/v0/Homo_sapiens_assembly38.dbsnp138.vcf
- Gold standard indels curated for recalibration: https://storage.googleapis.com/genomics-public-data/resources/broad/hg38/v0/Mills_and_1000G_gold_standard.indels.hg38.vcf.gz
- HapMap v3.3: https://storage.googleapis.com/genomics-public-data/resources/broad/hg38/v0/hapmap_3.3.hg38.vcf.gz
- OMNI 2.5: https://storage.googleapis.com/genomics-public-data/resources/broad/hg38/v0/1000G_omni2.5.hg38.vcf.gz
- 1000G phase 1 of known-site information: https://storage.googleapis.com/genomics-public-

data/resources/broad/hg38/v0/1000G_phase1.snps.high_confidence.hg38.vcf.gz
- Known indels curated for BQSR: https://storage.googleapis.com/genomics-public-data/resources/broad/hg38/v0/Homo_sapiens_assembly38.known_indels.vcf.gz
- dbNSFP 5.1a: https://www.dbnsfp.org/download
- gnomAD v4.1: https://gnomad.broadinstitute.org/downloads
- Clinvar: https://ftp.ncbi.nlm.nih.gov/pub/clinvar/vcf_GRCh38
- CADD v1.5: https://krishna.gs.washington.edu/download/CADD

6 **Reference Indexing**

Index the reference genome for downstream analysis:

```
cd ReferenceDir

# FASTA index
samtools faidx Homo_sapiens_assembly38.fasta

# Sequence dictionary required by GATK
gatk CreateSequenceDictionary \
  -R Homo_sapiens_assembly38.fasta \
  -O Homo_sapiens_assembly38.dict

# BWA-mem2 index
bwa-mem2 index Homo_sapiens_assembly38.fasta
```

Configure Nextflow Parameters

7 The GermVarX source code contains the following structure:

```
src/
   main.nf
   modules/
   pipeline/
   scripts/
nextflow.config
docker/
   germvarx-pipeline/
configuration/
   params.config
   docker.config
```

7.1 **src/**

Contains modularized workflow processes built according to the latest Nextflow structure. Processes are grouped into sub-workflows based on input type.

7.2 **nextflow.config**

Defines available custom profiles for execution.

7.3 **docker/**

Contains the Dockerfile for the GermVarX custom image.

7.4 **configuration/**

Contains configuration files:

- docker.config

Defines the Docker containers used by the pipeline.
1. Block 1: global container configuration
2. Block 2: process-specific container mapping

> Note
>
> When mounting directories using --volume, ensure the paths inside the container match local paths for simpler parameter configuration.

- params.config

Defines pipeline parameters (see Table 2).

	A	B
	**Parameter**	**Description**
	params.	Path to custom AB filter script.

	A	B
	py_ABfilter	
	params.outputDir	Output directory for all pipeline results.
	params.single_sample_mode	Run per-sample mode (true) or joint-calling mode (false).
	params.hard_filter	Apply GATK Hard Filters instead of VQSR.
	params.use_genomicsdb	Use GenomicsDB for joint-calling (instead of CombineGVCFs).
	params.inputDir	Directory containing FASTQ files.
	params.allFastq	Pattern to capture all FASTQ files.
	params.reads	Paired-end FASTQ pattern (_1 / _2 suffix).
	params.inputBAM	Input BAM file (if starting from aligned BAM).
	params.inputGVCF_gatk	Input GATK-generated GVCFs

	A	B
	params.inputGVCF_dv	Input DeepVariant-generated GVCFs.
	params.inputVCF_gatk	Input GATK VCF file
	params.inputVCF_dv	Input DeepVariant VCF file.
	params.output_type	If set, pipeline stops early and outputs only BAM or GVCF stage.
	params.readGroupLibrary	Read group library name (e.g., WES).
	params.readGroupPlatform	Sequencing platform (e.g., ILLUMINA).
	params.readGroupUnit	Read group unit (lane or flowcell ID).
	params.exomeRegionsBed	Target capture BED file for WES analysis.
	**Reference genome information**	

	A	B
	params.refDir	Directory containing reference genome resources.
	params.alignmentRef	Reference FASTA for alignment and variant calling.
	params.millsRef	Indel resource for BQSR and variant filtering.
	params.dbSNPRef	dbSNP reference VCF.
	params.hapmapRef	HapMap reference VCF for VQSR.
	params.omniRef	Omni reference VCF for VQSR.
	params.Ref1kG	1000 Genomes SNP reference.
	params.knownIndels	Known indel sites for BQSR.
	**Variant annotation resources**	
	params.vepCacheDir	VEP cache directory.
	params.plug	Directory containing annotation plugin resources.

	A	B
	insDir	
	params.vepPluginsDir	Subdirectory for VEP plugins.
	params.dbNSFP	dbNSFP annotation database.
	params.gnomAD	gnomAD exome frequency database.
	params.clinvar	ClinVar clinical variant database.
	params.caddIndel	CADD indel annotation resource.
	params.caddsnvs	CADD SNV annotation resource.
	**HardFilter thresholds (if hard_filter = true)**	
	params.indelQUAL	Hard filter: minimum QUAL for indels.
	params.indelINFO_QD	Hard filter: Quality by Depth (QD) for indels.
	params.indelINFO_Read	Hard filter: Read position bias for indels.

	A	B
	PosRankSum	
	params.indelINFO_FS	Hard filter: Fisher strand bias for indels.
	params.snpQUAL	Hard filter: minimum QUAL for SNPs.
	params.snpINFO_QD	Hard filter: Quality by Depth (QD) for SNPs.
	params.snpINFO_MQ	Hard filter: Mapping Quality for SNPs.
	params.snpINFO_ReadPosRankSum	Hard filter: Read position bias for SNPs.
	params.snpINFO_MQRankSum	Hard filter: Mapping Quality Rank Sum for SNPs.
	params.snpINFO_FS	Hard filter: Fisher strand bias for SNPs.
	params.snpINFO	Hard filter: Strand Odds Ratio for SNPs.

	A	B
	_SOR	
	**Cohort-Level Variant Quality Control thresholds**	
	params.QUAL	Minimum QUAL that a variant is truly polymorphic across the cohort.
	params.DP	Genotypes with sequencing depth below this threshold are set to missing.
	params.GQ	Genotypes with genotype quality below this threshold are set to missing.
	params.ABlower	Heterozygous calls with allele balance below this threshold are set to missing.
	params.ABupper	Heterozygous calls with allele balance above this threshold are set to missing.
	params.VarCallRate	Variant call rate across the cohort.
	**Parallelism (forks)**	
	params.heavyFork	Number of parallel jobs for heavy tasks (alignment, variant calling).
	params.lightFork	Number of parallel jobs for lightweight tasks (QC, annotation).

Table 2: GermVarX – Pipeline Parameters

Note

⚠️ *Important:* Certain parameters should not be defined directly in params.config. Instead, these input paths must be provided as command line arguments. Refer to Below Section for further details.

Run the Pipeline

8 After parameter configuration, run the pipeline from the GermVarX directory (where nextflow.config is located):

```
nextflow run src/main.nf -profile docker  [OPTIONS]
```

To run from another directory:

```
nextflow run /path/to/project/src/main.nf \
  -c /path/to/project/nextflow.config \
  -profile docker  [OPTIONS]
```

INPUT Options:

- **FASTQ input**

```
nextflow run src/main.nf -profile docker --inputDir
<path/to/folder_fastq_files>
```

- **BAM input**

```
nextflow run src/main.nf -profile docker --inputBAM
<path/to/folder_BAM_files>
```

- **GVCF input (GATK)**

```
nextflow run src/main.nf -profile docker --inputGVCF_gatk
<path/to/folder_GATK_GVCF_files >
```

- **GVCF input (DeepVariant)**

```
nextflow run src/main.nf -profile docker --inputGVCF_dv
<path/to/folder_DeepVariant_GVCF_files>
```

- **VCF input (GATK)**

```
nextflow run src/main.nf -profile docker --inputVCF_gatk
<path/to/folder_GATK_VCF_files >
```

- **VCF input (DeepVariant)**

```
nextflow run src/main.nf -profile docker --inputVCF_dv
<path/to/folder_DeepVariant_VCF_files>
```

- **FASTQ input and GVCF output**

```
nextflow run src/main.nf -profile docker --inputDir
<path/to/folder_fastq_files> --output_type GVCF
```

Optional Parameters:

	A	B	C
	**Parameter**	**Description**	**Default**
	-work-dir <workspace_directory>	Path to intermediate files	work
	--outputDir<br><br><path/to/outputDir>	Path to final results	output
	--output_type {BAM | GVCF}	Stop pipeline early at BAM or GVCF stage	null (full pipeline)
	--use_genomicsdb	Use GenomicsDBImport instead of CombineGVCFs	FALSE
	--hard_filter	Apply hard filters instead of VQSR (recommended for small sample sets)	FALSE

	A	B	C
	--single_sample_mode	Run in single-sample mode (no joint genotyping)	FALSE

Illustrative test case

9 To demonstrate the execution of **GermVarX**, we provide an example of runing the full pipeline on FASTQ data located in the testdata/fastq directory (see Step 4). From the GermVarX root directory (where nextflow.config is located), the full pipeline can be executed with the following command:

```
nextflow run src/main.nf -profile docker \
--inputDir <path/to/testdata/fastq> \
--outputDir out_testfullpipe
```

Before execution, users should ensure that all parameters are properly configured, reference paths are correctly specified, and the BED file provided in testdata/bed is included. Upon successful completion, the execution report will be displayed on the terminal (see Figure 1).

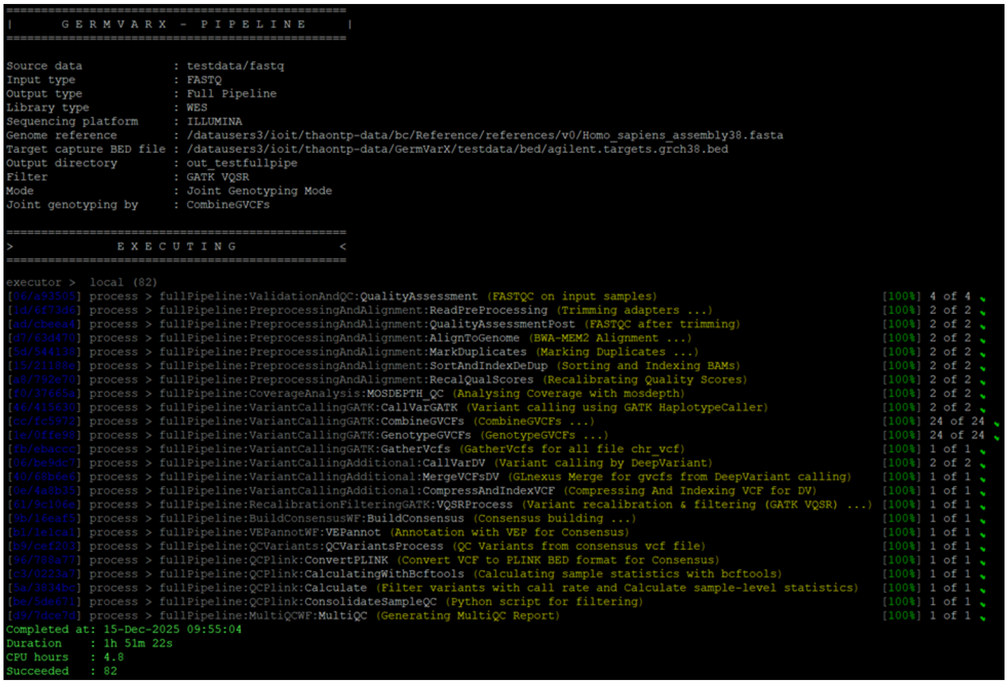

Figure 1: Screenshot of the GermVarX execution report and completion summary displayed on the terminal.

9.1 The output directory out_testfullpipe will contain the final results, organized into multiple subfolders (see Figure 2) and associated files corresponding to each stage of the pipeline.

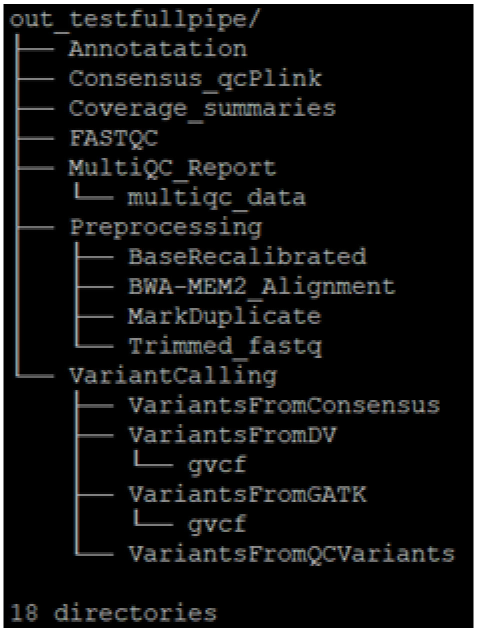

Figure 2: Directory structure of out_testfullpipe.

9.2 **Example with Hard Filtering**

To run the workflow with hard filtering enabled (instead of the default VQSR), use the following command:

```
nextflow run src/main.nf -profile docker \
--inputDir  <path/to/testdata/fastq> \
--outputDir out_hardfilter --hard_filter
```

In this case, the terminal output will reflect the hard filtering process, as illustrated in Figure 3.

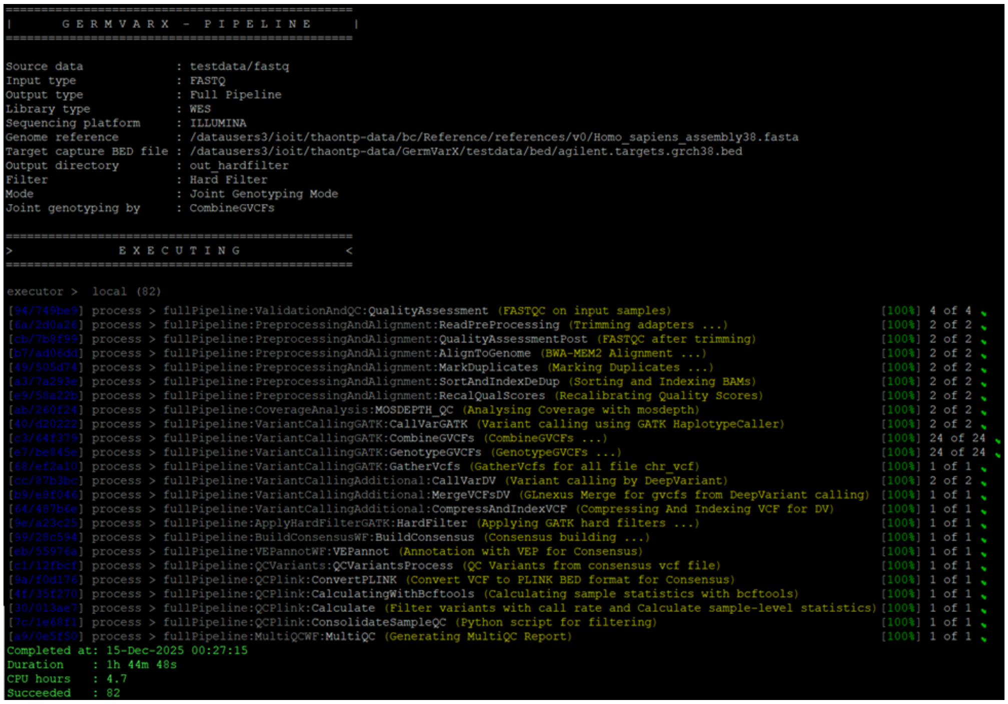

Figure 3: Screenshot of the GermVarX execution report and completion summary displayed on the terminal with hard filtering enabled.
