# Supplementary material for: GermVarX: A Robust Workflow for Joint Germline Variant Exploration in whole-exome sequencing cohorts
Source: PLoS One. 2026 Apr 2;21(4):e0345561. doi: 10.1371/journal.pone.0345561 (PMC13046259; doi:10.1371/journal.pone.0345561)
Supplement: Attachment — Submitted filename: ResponsetoReviewers.docx [file pone.0345561.s003.docx]

Dear editor and reviewers,

We appreciate your comments and suggestions. We addressed all of your comments and made changes directly in the manuscript where appropriate. Please find our detailed responses to the reviewers below.

Best Regards,

Thao Nguyen

**Reviewer #1:**

My main concern is that the described pipeline appears to be primarily a combination of existing software tools, which raises questions about its novelty and added value. The authors are encouraged to provide a stronger justification for the development of this pipeline—specifically, to clarify what unique features, integrations, or improvements it offers compared to existing solutions, and to articulate how it will advance or benefit the research community.

The authors state in the introduction: “While a few pipelines such as MagicPipeline [5] and star protocols wes [6] support multi-sample processing, they are predominantly script-based and command-line dependent, lacking key features such as automated orchestration, modular parallel processing, and scalability across diverse computing infrastructures.” However, it remains unclear how the proposed pipeline substantively improves upon these existing tools. Furthermore, many researchers working with WES data already employ well-established, in-house pipelines tailored to their specific workflows. As such, the practical advantages and broader relevance of the proposed pipeline to the wider research community are not evident.

We appreciate the reviewer’s concern about the pipeline’s novelty. As noted, modern bioinformatics pipelines inevitably rely on established, high-performance tools. As with other widely used workflows mentioned in the Introduction (e.g., Sarek, DNAscan2), GermVarX does not introduce new algorithms. Instead, its novelty and value stem from the **engineering, integration, and optimization of a fully automated, reproducible, and high-confidence cohort-scale workflow specifically tailored for germline WES data**.

From a user’s perspective, many publicly available datasets (e.g., case–control studies) are distributed as raw FASTQ files, requiring substantial preprocessing before downstream analyses are possible. Although several tools support individual components of WES cohort processing, they typically do not offer the integrated set of features provided by GermVarX, including automated workflow orchestration, modular parallelization, full containerization, reproducibility, portability, and scalability. Moreover, **GermVarX has been quantitatively validated for accuracy on a benchmark dataset, a level of evaluation not available for other WES cohort workflows, such as MagicPipeline or star_protocols_wes.** GermVarX therefore offers a practical, ready-to-use solution that eliminates the considerable effort required for researchers to develop, validate, and maintain an equivalent in-house script-based workflow. We believe this integration directly benefits the broader research community, particularly groups without dedicated bioinformatics engineering support.

We have revised the Introduction section (highlighted in blue in the manuscript) to articulate better the motivation and context for developing GermVarX. In addition, we have included **a detailed comparison table (Table 3)** in the Discussion section (new text at lines 261-264), which summarizes the key differences between GermVarX and several existing WES workflows.

Below are several additional comments and questions regarding the manuscript.

1) Authors use a 3-sample WES benchmark dataset for validation, but it would be more informative if they could conduct validations on additional data sets.

Our current validation includes two complementary use cases designed to demonstrate both accuracy and scalability:

- **3-sample WES GIAB benchmark:** We used the well-known Ashkenazim Trio dataset, which has an established variant truth set. This allowed us to perform an objective, quantitative measurement of core accuracy metrics (Precision, Recall, and F1-score) for our three primary calling strategies (GATK-Joint, DeepVariant-GLnexus, and the Consensus set) against a gold standard. This level of granular accuracy benchmarking is not possible with most large public datasets.
- **120-sample real WES cohort:** used to assess practical performance, robustness, and scalability in a real-world cohort setting.

GermVarX was designed following current best practices and up-to-date guidelines for WES cohort processing. During development, we considered the challenge of merging large numbers of per-sample GVCFs into a combined cohort GVCF and therefore included **support for GenomicsDBImport as a scalable alternative to CombineGVCFs.** Although this option was already available and described in the Optional Parameters section of the S1_File, we inadvertently omitted mentioning it in the manuscript. This has now been corrected in the revised version (Lines 100-104).

We also observed that the merging process is time-consuming and that GATK does not support parallelization for the CombineGVCFs / GenomicsDBImport and GenotypeGVCFs steps. To address this, **GermVarX performs per-chromosome merging and joint genotyping**, enabling efficient parallel execution and substantially reducing runtime. This technical improvement explains the 24 parallel processes utilized for both CombineGVCFs and GenotypeGVCFs shown in Figures 1 and 3 of the S1_File. The manuscript has also been updated to clearly indicate this functionality (new text at lines 284–287).

While we have established accuracy and moderate-scale performance, we agree that incorporating additional, larger, and independent datasets would further strengthen the manuscript. However, due to immediate resource and data-access constraints, such large-scale external validation could not be included in the current version. We incorporated this as part of our future work, including benchmarking on publicly available large-scale cohorts to further stress-test scalability and extending the evaluation to whole-genome sequencing (WGS) data to demonstrate the pipeline's flexibility beyond WES (Lines 301-303).

2) On page 6, they state: "the highest 185 F1-score (0.9649), balancing exceptional precision (0.9994) with strong recall (0.9327). Please clearly define these 3 metrics.

Thank you for pointing this out. We have added clear definitions of **precision**, **recall**, and **F1-score** immediately before this paragraph (Lines 210–220).

3) Figure 2 needs a legend.

Thanks. We have added a complete legend for Figure 2 in the revised manuscript.

4) In the subsection "Variant Filtering and Quality Control" on page 5, are users able to modify the filtering thresholds? For example, instead of DP < 10, can the users change it to DP < 20? This was not very clear.

We appreciate the reviewer’s question. We have updated the workflow to allow users to **easily modify all filtering thresholds** (e.g., DP, GQ, AB) directly through the configuration/params.config file. The manuscript has also been updated to clearly indicate this functionality (Lines 130–131). In addition, a detailed description of these parameters has been included in ***Table 2: GermVarX – Pipeline Parameters*** in the S1_File.

5) Can authors also explain if their pipeline can handle if the users input the intermediate files? For example, if the users already have gVCF files, can the pipeline bypass the previous steps and start with the gVCF files?

Yes, our pipeline is fully capable of handling intermediate files, including gVCF files, and can bypass previous steps.

As stated at the end of the “Setting up and running GermVarX pipeline” section of our manuscript:" By default, GermVarX executes the full pipeline, beginning with paired-end FASTQ files and proceeding through alignment, variant calling, filtering, annotation, and reporting. **However, the workflow also supports alternative entry points, accepting BAM, GVCF, or VCF files as input. In such cases, execution commences automatically at the appropriate downstream stage, thereby bypassing earlier processes.** Similarly, users can specify the desired final output format (e.g., BAM, GVCF), allowing the pipeline to terminate at a chosen stage."

If a user provides gVCF files, GermVarX automatically skips the Per-Sample Processing phase and proceeds directly to the cohort-level joint genotyping steps (GATK CombineGVCFs/ GenomicsDBImport and GATK GenotypeGVCFs or GLnexus), then continues with filtering and annotation. Full details on specifying these entry points and configuration options are provided in the Run the Pipeline section of the S1_File.

6) A major improvement to the current manuscript would be to demonstrate that the pipeline can handle two or more cohort-level WES datasets originating from different sources and generate a merged, harmonized dataset. This is a technically challenging task, and successfully implementing such functionality would significantly enhance the novelty and impact of the work. However, it is acknowledged that this capability may be beyond the current scope of the study.

Thank you for this valuable suggestion. In practice, merging and harmonizing WES datasets from different sources is typically performed to enrich downstream analyses or to support tasks such as estimating population structure and sample ancestry. Such merged cohort datasets are generally constructed from GVCF/VCF files, because processing raw sequencing data across multiple cohorts would require prohibitive storage and computational resources. Importantly, GVCF/VCF merging requires strict consistency in the reference genome, upstream processing steps, and variant-calling parameters to minimize technical batch effects. The challenges and considerations involved in combining WES datasets from multiple sources are well described in the literature (e.g., Briefings in Bioinformatics, 2016; [https://academic.oup.com/bib/article/17/4/672/2240552](https://academic.oup.com/bib/article/17/4/672/2240552?utm_source=chatgpt.com)).

At present, GermVarX is designed to generate a reproducible, high-confidence multi-sample VCF for a single, well-defined cohort, ensuring uniform processing and reducing variability arising from differences in sequencing protocols or analysis pipelines. Extending the workflow to handle merged, harmonized datasets from disparate sources would require substantial cohort-level quality control and harmonization steps. This capability is not within the current scope of the initial release but is a high-priority feature for future development. As noted in the final paragraph of our Discussion section, we are already planning these enhancements: “Future optimizations could include support for storage-efficient formats such as CRAM, **as well as** **automated cohort-level quality control steps (e.g., sample filtering, PCA, ancestry inference, population stratification adjustment)**. Such extensions would further expand GermVarX beyond variant discovery toward a comprehensive population genomics and translational research platform.”

We fully agree that supporting multi-cohort harmonization would significantly increase the novelty and impact of the workflow. This is also our concern when working with Vietnamese WES datasets, where cohort sizes are modest and combining external WES datasets is often necessary to increase analytical power. **By first delivering a highly reliable and reproducible single-cohort workflow, we establish a clean, standardized foundation on which a robust multi-cohort harmonization module can be efficiently developed.**

**Reviewer #2:**

1) The authors state that existing workflows are designed for single-sample analysis, while their workflow is optimized for multi-sample analysis (L13). However, one of the existing workflows they cite, Sarek, states in their documentation that it is very easy to run multiple samples via a CSV samplesheet. It seems to me that the authors should not emphasize single vs multi sample design, but rather their implementation of joint calling.

Thank you for your comment. We have revised the Introduction section (highlighted in blue in the manuscript) to better articulate the motivation and context for developing GermVarX.

2) Related to above, I believe that the authors could expand more on how their workflow is different than the existing workflows they listed, and why those differences are important. As it is written currently, the only real distinction the authors make is the difference in design for single or multi sample analysis, which is insufficient. The authors could consider making a table to list the differences to make it easier for readers (i.e. potential users) to determine if this workflow is right for their application.

Thank you for your suggestion. We have added a **comparison table (Table 3)** in the Discussion section (new text at lines 261-264), which summarizes the key differences between GermVarX and several existing WES workflows.

In constructing this table, we selected **Sarek** as the representative per-sample processing workflow because it is an actively maintained, community-driven, open-source pipeline built on Nextflow DSL2. Sarek is supported by a large and active developer community (79 contributors as of 10 December 2025) and receives continuous updates and optimizations. For completeness, we have also updated the Sarek citation to reflect its most recent peer-reviewed publication.

**MagicPipeline** and **star_protocol_wes** are included in the table because, to our knowledge, they are the only publicly available pipelines specifically designed for WES cohort studies.

3) The workflow Github repo looks well documented, for which I commend the authors. I would also like to see the inclusion of a test dataset that users can run through the workflow to make sure the workflow runs correctly on their system.

Thank you for the suggestion. We have now included a link to a test dataset that users can download and run through GermVarX to verify correct installation and execution. If the paper is accepted, we will update the repository with a citation to the publication, which includes a fully detailed guideline in the S1_file.

**Reviewer #3:**

1. Add runtime comparisons with other public workflows. A qualitative comparison will strengthen the claim of scalability.

We understand the value of runtime comparisons. However, a direct and quantitative head-to-head comparison with other public workflows is challenging for several reasons: (i) publicly available pipelines differ in configuration, software versions, and hardware assumptions; and (ii) the two available cohort-level WES protocols (MagicPipeline and STAR Protocol WES) are script-based and do not support parallelization, making runtime comparisons inherently unfair relative to a fully orchestrated workflow such as GermVarX.

To provide meaningful context, we performed a **qualitative comparison using Sarek**, a well-maintained, open-source workflow built on Nextflow DSL2. Running Sarek on the GIAB Ashkenazim Trio (3 WES samples) required approximately **6 hours 22 minutes**, which is slightly faster than GermVarX’s runtime of **7 hours 03 minutes** when using GATK Hard Filtering. This result highlights that GermVarX operates within a comparable timeframe while performing additional cohort-level steps that Sarek does not include.

GermVarX was designed following current best practices and up-to-date guidelines for WES cohort processing. During development, we considered the challenge of merging large numbers of per-sample GVCFs into a combined cohort GVCF and therefore included support for **GenomicsDBImport as a scalable alternative to CombineGVCFs**. Although this option was already available and described in the Optional Parameters section of the Supplementary Information (S1_File), we inadvertently omitted mentioning it in the manuscript. This has now been corrected in the revised version (Lines 100-104).

We futher observed that GATK does not support parallelization for the CombineGVCFs / GenomicsDBImport and GenotypeGVCFs steps. To address this, GermVarX performs **per-chromosome merging and joint genotyping**, enabling efficient parallel execution and substantially reducing runtime. This technical improvement explains the 24 parallel processes utilized for both CombineGVCFs and GenotypeGVCFs shown in Figures 1 and 3 of the S1_File. The manuscript has also been updated to clearly indicate this functionality (Lines 284–287).

We also provide strong, quantitative evidence of GermVarX’s performance on a real 120-sample WES cohort (403 GB of raw FASTQ data). The workflow completed processing—from FASTQ to annotated joint VCF—in **less than 78 hours** on a mid-range compute cluster. This result qualitatively supports our claim of robust scalability for WES cohort studies.

Due to immediate resource and data-access constraints, large-scale external validation could not be included in the current version. We incorporated this into our future work, including benchmarking on publicly available large-scale cohorts to further stress-test scalability and extending the evaluation to whole-genome sequencing (WGS) data to demonstrate the pipeline's flexibility beyond WES (Lines 301-303).

2. Future work could include the integration of structural variant and copy number variation (CNV) detection modules, along with extending the workflow to accommodate whole-genome sequencing (WGS) and long-read data for comprehensive variant discovery. This would enhance its applicability to diverse genomic analyses.

Thank you for your suggestion. We have added these points to the future work (new text at lines 301-308).

3. Figure captions could be expanded to interpret the figure rather than reviewing the section text.

Thanks. We have updated the figure captions to incorporate additional interpretative descriptions.
